# First Findings From PLAN: Multi-Site RCT for Korean American Older Adults With Undiagnosed Dementia and Caregivers

**DOI:** 10.1093/geroni/igaf122.362

**Published:** 2025-12-31

**Authors:** Hae-Ra Han, Nancy Perrin, Ji-Young Yun, Deborah Min, Simona Kwon, Jin hui Joo, Hochang Lee

**Affiliations:** Johns Hopkins University, Baltimore, Maryland, United States; Johns Hopkins University, Baltimore, Maryland, United States; Johns Hopkins University, Wellesley, Massachusetts, United States; Johns Hopkins Bloomberg School of Public Health, United States; New York University, New York, New York, United States; Massachusetts General Hospital, Boston, Massachusetts, United States; University of Rochester, Rochester, New York, United States

## Abstract

Linking individuals to medical services is critical for early dementia detection, diagnosis, and care. Racial and ethnic minoritized older adults, particularly those with limited English proficiency (LEP), face significant barriers to dementia evaluation and care. Older Korean Americans (KAs)—one of the fastest-growing aging populations in the US—are predominantly monolingual first-generation immigrants, making them especially vulnerable to dementia inequities. Community health workers (CHWs) can help bridge linguistic and health literacy gaps. We evaluated the effectiveness of PLAN (Preparing for healthy aging through dementia Literacy education And Navigation), a CHW-led intervention promoting dementia care access, in a multi-site clinical trial across the Greater Washington and New York metropolitan areas. Using a two-step eligibility screening with Mini-Mental State Exam (n = 2,698) followed by Clinical Dementia Rating (CDR, n = 393), a total of 288 dyads of KA older adults with undiagnosed dementia (CDR 1+; mean age=83, 73% women) and their caregivers (mean age=67, 69% women) were enrolled and followed for six months. Results showed a significantly higher rate of linkage to medical services in the intervention group compared to the control group (16.5% vs. 0%, X2 [1]=23.69, p<.001). No significant differences were observed in secondary outcomes, including caregiver dementia knowledge, self-efficacy, depression, and quality of life. To our knowledge, this is the first study to test the efficacy of a CHW-led, community-based transition model for dementia evaluation and care in an older LEP population. Findings highlight the effectiveness of PLAN in facilitating dementia care access for underserved immigrant communities facing significant language barriers.

